# Knockout of NOS2 Promotes Adipogenic Differentiation of Rat MSCs by Enhancing Activation of JAK/STAT3 Signaling

**DOI:** 10.3389/fcell.2021.638518

**Published:** 2021-03-19

**Authors:** Aiping Qin, Sheng Chen, Ping Wang, Xiaotao Huang, Yu Zhang, Lu Liang, Ling-Ran Du, De-Hua Lai, Li Ding, Xiyong Yu, Andy Peng Xiang

**Affiliations:** ^1^Key Laboratory of Molecular Target and Clinical Pharmacology, State Key Laboratory of Respiratory Disease, The Fifth Affiliated Hospital, School of Pharmaceutical Sciences, Guangzhou Medical University, Guangzhou, China; ^2^Center for Stem Cell Biology and Tissue Engineering, Key Laboratory for Stem Cells and Tissue Engineering, Ministry of Education, Sun Yat-sen University, Guangzhou, China; ^3^Center for Parasitic Organisms, State Key Laboratory of Biocontrol, School of Life Sciences, Sun Yat-sen University, Guangzhou, China; ^4^Department of Pathology, The First Affiliated Hospital, Sun Yat-sen University, Guangzhou, China

**Keywords:** rat mesenchymal stromal cells, NOS2, adipogenesis, differentiation, JAK/STAT3 signaling

## Abstract

Mesenchymal stromal cells (MSCs) are a heterogeneous population of cells that possess multilineage differentiation potential and extensive immunomodulatory properties. In mice and rats, MSCs produce nitric oxide (NO), as immunomodulatory effector molecule that exerts an antiproliferative effect on T cells, while the role of NO in differentiation was less clear. Here, we investigated the role of NO synthase 2 (NOS2) on adipogenic and osteogenic differentiation of rat MSCs. MSCs isolated from NOS2-null (NOS2^–/–^) and wild type (WT) Sprague–Dawley (SD) rats exhibited homogenous fibroblast-like morphology and characteristic phenotypes. However, after induction, adipogenic differentiation was found significantly promoted in NOS2^–/–^ MSCs compared to WT MSCs, but not in osteogenic differentiation. Accordingly, qRT-PCR revealed that the adipogenesis-related genes PPAR-γ, C/EBP-α, LPL and FABP4 were markedly upregulated in NOS2^–/–^ MSCs, but not for osteogenic transcription factors or marker genes. Further investigations revealed that the significant enhancement of adipogenic differentiation in NOS2^–/–^ MSCs was due to overactivation of the STAT3 signaling pathway. Both AG490 and S3I-201, small molecule inhibitors that selectively inhibit STAT3 activation, reversed this adipogenic effect. Furthermore, after high-fat diet (HFD) feeding, knockout of NOS2 in rat MSCs resulted in significant obesity. In summary, NOS2 is involved in the regulation of rat MSC adipogenic differentiation *via* the STAT3 signaling pathway.

## Introduction

Mesenchymal stromal cells (MSCs) are self-renewing multipotent stromal cells that can be isolated from mesenchymal tissues, such as bone marrow, adipose tissue, dental pulp, umbilical cord blood, and other tissues. MSCs are defined according to their lack of the hematopoietic and endothelial markers CD45 and CD34 and their expression of the stromal markers CD105, CD90, CD29, CD44, and CD73 (Friedenstein et al., 1970; Zuk et al., 2001). MSCs have drawn significant attention from the research community with their ability to exert suppressive and regulatory effects on both adaptive and innate immunity, as demonstrated by recent studies (Bernardo and Fibbe, 2013; Li and Hua, 2017). The therapeutic potential of MSCs is attributed to complex cellular and molecular mechanisms of action, including regulation of immune responses *via* immunomodulation and differentiation into multiple cell lineages.

*In vitro*, MSCs are capable of differentiating into cartilage, bone, tendon, adipose tissue, muscle, etc. Among these tissues, MSCs are commonly progenitors of osteoblasts and adipocytes (Pittenger et al., 1999). Interestingly, the relationship between osteogenesis and adipogenesis in the bone marrow seems to be reciprocal, as stimulation of MSC osteogenesis occurs at the expense of adipogenesis (Chen et al., 2016; Li et al., 2019). Understanding the signaling pathways and regulators that govern MSC osteogenic and adipogenic differentiation may improve the appreciation of MSC functions and holds great promise for the application of cell therapy and regenerative medicine, particularly from the viewpoint of developing new therapeutic treatments for bone loss. However, the precise mechanisms that regulate the differentiation of MSCs and determine the fate of stem cells are still unclear (Pittenger et al., 1999; Discher et al., 2009; Chen et al., 2016; Li et al., 2019).

Studies on embryonic and adult stem cells suggest that many biological processes, including self-renewal, viability, migration, proliferation and differentiation, are regulated by nitric oxide (NO). Indeed, to a great extent, NO production also largely influences MSC survival, homing and even differentiation (Ren et al., 2008; Bonafè et al., 2015; Wang et al., 2015). NO is an endogenous molecule produced by NO synthases (NOSs) through a complex oxidoreductase reaction that consumes L-arginine and oxygen. There are three types of NOSs, inducible NOS (NOS2), NOS1, and NOS3. NOS1 and NOS3 are constitutively expressed in neurons and epithelial cells and produce low (nM) levels of NO in a process that is regulated by Ca^2+^ binding to calmodulin (Alderton et al., 2001). In contrast, NOS2 is only induced by immunological stimuli in a calcium-independent manner and produces large amounts of NO (μM levels) (Nathan, 1992; Moncada and Higgs, 1993).

The production of NO by NOS2 may be applied in mouse and rat MSCs to inhibit T cell proliferation (Ren et al., 2008; de Castro et al., 2019). However, the roles of NO in T cell-mediated immunity, inflammation and tumor growth and metastasis remain debated, and the lack of clarity may be due to variance in NO concentration or NO sources (endogenous or exogenous) (Vannini et al., 2015; Garcia-Ortiz and Serrador, 2018). Therefore, this debate requires an update with new data. In addition, whether and how NOS2 plays a role in MSC differentiation remains largely unknown.

In this study, using NOS2-null (NOS2^–/–^) rats, we found that MSCs without NOS2 significantly shifted toward adipogenic differentiation. The clear association between NOS2 and adipogenic differentiation in bone marrow-derived MSCs (BMSCs) was mediated by the STAT3 signaling pathway. This study provides deeper insights into the regulation of MSC adipogenic differentiation and presents evidence for new roles played by NOS in this process.

## Materials and Methods

### Animals

As previously described (Shen et al., 2017), wild type (WT) and NOS2^–/–^ Sprague–Dawley (SD) rats were used in this study. Rats were housed in a specific pathogen-free facility and were given free access to food and water. All animal procedures were performed under the supervision of the Experimental Animal Ethics Committee of Guangzhou Medical University (Guangzhou, China).

To observe osteogenesis and fat formation, eight-week-old female WT and NOS2^–/–^ rats were randomly divided into two groups and fed either a normal chow diet (NCD) or a high-fat diet (HFD) (D12492, Research Diets, New Brunswick, NJ, United States) for 8 weeks and weighed weekly until sacrifice. Bone mineral content (BMC) and bone mineral density (BMD) were measured by dual-energy X-ray absorptiometry (DXA), and adipose tissues were collected for HE staining and western blot assay.

### Isolation and Cultivation of Rat MSCs

Bone marrow-derived MSCs were isolated from femurs and tibias of NOS2^–/–^ and WT SD rats under aseptic conditions. Briefly, femurs and tibias were dissected and soaked in cold PBS. The bone marrow was exposed and flushed with 4–5 ml cell culture medium. BM cells, including hematopoietic stem cells and marrow stromal cells, were cultured in low-glucose Dulbecco’s modified Eagle’s medium (DMEM) (Thermo Fisher Scientific, Waltham, MA, United States) containing 15% FBS at 37°C and 5% CO_2_. After 3 days in culture, non-adherent cells were washed off, and adherent MSCs were cultured in medium that was replaced every 3 days for 10–14 days, at which time cells had formed homogenous fibroblast-like colonies. MSC colonies were further passaged, and passages 3–5 were used in subsequent assays. Flow cytometry analysis was used to assess MSCs and their multipotent properties.

Adipose-derived MSCs (AdMSCs) were isolated from the inguinal area of rats, washed several times with HBSS, and minced with scissors. Then, 0.1% type I collagenase in HBSS without Ca^2+^ or Mg^2+^ was added, and samples were incubated for 60 min at 37°C, followed by a wash and resuspension in HBSS. After filtering through a 70 μm cell filter and washing twice, cells were pelleted, resuspended in MSC culture medium, and then plated in a 25 cm^2^ dish. MSCs were isolated, passaged and quantified as described above.

### Flow Cytometry

To identify cell surface markers, a BMSC suspension (1 × 10^6^ cells/mL) was prepared and washed twice with PBS and then incubated in the dark for 40 min at 4°C with monoclonal antibodies (mAb) against α-CD34-PE (188-10041, RayBiotech, United States), α-CD29-FITC (Clone: Hα2/5), α-CD45-FITC (Clone:OX-1), α-CD90-PE (Clone:OX-7) (BD Biosciences, Franklin Lakes, NJ, United States) and their corresponding isotype antibodies. Stained cells were subsequently rinsed with cold PBS and immediately subjected to flow cytometry analysis (BD LSRII; BD Biosciences) to determine cell surface marker expression.

### T Cell Proliferation Assays

T cell proliferation was evaluated by CellTrace CFSE (5,6-carboxyfluorescein diacetate, succinimidyl ester) cell proliferation kit (Invitrogen, Carlsbad, CA, United States). Briefly, single-cell suspensions of WT rat spleens were prepared and stained with CD3-APC (Clone: 1F4) for 40 min at 4°C. CD3 + T cells were isolated by flow cytometry sorting (BD LSRII; BD Biosciences) and were then labeled with 5 mM CFSE. The labeled T cells were cultured in replicate wells with or without previously plated BMSCs at a 20:1 ratio and were stimulated with α-CD3 (0.5 mg/mL) and α-CD28 (1 mg/mL) mAbs (EBioscience, San Diego, CA, United States) for 96 h. Cells were then stained for surface marker expression with CD4-PE (Clone: OX-38) and CD8α-PerCP (Clone: OX-8) antibodies (BD Biosciences) and incubated 40 min at 4°C. T cell proliferation was detected by flow cytometry.

### Cell Survival and Proliferation Assays

MSC survival was determined by plating 1 × 10^5^ BMSCs per well in six-well plates. After overnight incubation, media were replaced with serum-free DMEM, and cells were cultured for an additional 48 h. Cell survival was determined using an Alexa Fluor 488 Annexin V/PI Kit (Invitrogen) according to the manufacturer’s instructions. The ratios of dead to cells alive were determined from five randomly selected 40x microscopic fields.

A cell proliferation assay was performed to determine the population doubling time (PDT) of NOS2^–/–^ and WT BMSCs using a fluorescence-based Cell Counting Kit-8 (CCK8) cell proliferation assay (Dojindo, Kumamoto, Japan). In brief, 100 μl of BMSC suspension was plated onto a 96-well plate at 2,500 cells per well and cultured for 24 h, after which 10 μl of CCK8 solution was added to each well and the plate was incubated for 1–4 h. Cell proliferation was assessed at 450 nm using a microplate reader (Tecan Trading AG, Switzerland).

### Multilineage Differentiation *in vitro*

Cells were induced to adipogenic and osteogenic differentiation in culture. All differentiation cultures received regular MSC expansion medium (Ren et al., 2008; Qin et al., 2017).

For adipogenic differentiation, MSCs derived from rats were seeded into 6-well or 12-well plates (Nest, China). When confluence reached 80–90%, cells were cultured in adipogenic differentiation medium (AM A or AM B) (Cyagen BioSciences, United States) for 12 days. AM A was made from AM B by adding 1 μM dexamethasone, 0.2 mM indomethacin and 0.5 mM isobutylmethylxanthine. AM B was composed of DMEM plus 10% FBS, 1% penicillin-streptomycin, 1% glutamine and 0.01 mg/mL insulin. BMSCs were maintained in AM A for 3 days, AM B for 1 days, AM A for 3 days, AM B for 1 days, AM A for 3 days and AM B for 1 days to promote adipocyte differentiation. Lipid content was evaluated with Oil Red O staining (Sigma, St. Louis, MO, United States) after cells were fixed in 4% PFA. Images were acquired using a phase contrast light microscope (Leica Dmi8) (Leica Microsystems, Germany).

For osteogenic induction, BMSCs were seeded into 6-well or 12-well plates. When BMSCs reached a density of 80–90%, cells were grown for 21 days in osteogenic medium (OM) (Cyagen Biosciences), which contained 10% FBS, 1% glutamine, 0.2% ascorbic acid, 1% penicillin-streptomycin, 0.01% dexamethasone, and 1% β-glycerophosphate to promote osteoblast induction. After fixation, cells were analyzed by staining with Alizarin red (Sigma-Aldrich) as previously reported (Qin et al., 2017). Experiments for each group were conducted in triplicate wells.

### NO Detection

Supernatants of MSC differentiation and activated T/MSCs cocultures were collected and stored at −80°C. The NO concentration was assessed following the manufacturer’s protocol (Beyotime, China).

### RNA Extraction and Quantitative Real-Time PCR Analysis

Total cellular RNA was extracted using TRIzol reagent (Invitrogen). A NanoDrop spectrophotometer (Thermo Fisher Scientific) was utilized to analyze the quantity and quality of the RNA. First-strand cDNA was synthesized from 1 μg RNA according to the instructions of the TaKaRa reverse transcription kit. The mRNA levels of the indicated genes were analyzed in triplicate using QuantiTect SYBR Green PCR Master Mix (Takara, Kyoto, Japan). Reactions were run on a LightCycler480 real-time PCR instrument (Roche, Indianapolis, IN, United States). The gene expression levels were normalized to GAPDH. The relative expression of target genes was assessed by the 2^–Δ^ ^Δ^ ^*Ct*^ method. Specific primers for rat PPAR-γ, C/EBP-α, FABP4, LPL, ALP, RUNX2, COL1A1, and GAPDH are listed in Supplementary Table 1.

### Western Blotting

Quantitative analysis of changes in protein expression was conducted by western blot analysis according to previous reports (Qin et al., 2017). BMSCs were inoculated into 6-well plates and differentiated when cells reached 80% confluence. After induction, cells were washed twice with precooled PBS, lysed in radioimmunoprecipitation assay (RIPA) lysis buffer (Thermo Fisher Scientific) at 4°C for 30 min, sonicated for 30 s, and centrifuged at 12,000 g for 20 min. The resulting supernatants were collected, and protein concentrations were measured using a bicinchoninic acid protein assay kit (Sigma-Aldrich). Total protein was separated by SDS-PAGE and transferred to PVDF membranes. The membranes were blocked in 5% non-fat milk (in Tris-buffered saline containing 0.1% Tween-20) for 1.5 h and then incubated with primary antibodies [α-phosphorylated (p)-STAT1 (1:1,000, #7649), α-p-STAT3 (1:1,000, #9145), α-p-STAT 5 (1:1,000, #4322), α-STAT1 (1:1,000, #14994), α-STAT3 (1:1,000, #9139), α-STAT 5 (1:1,000, #94205), α-GAPDH (1:1,000, #5174), α-p-JAK2 (1:1,000, #3776), α-JAK2 (1:1,000, #3230) from Cell Signaling Technology (Danvers, MA, United States) and α-PPAR-γ (1:500, #ab209350), α-NOS2 (1:500, #ab3523) from Abcam (Cambridge, MA, United States)] and then with a horseradish peroxidase-conjugated secondary antibody for 1 h at room temperature. Finally, blots were digitally processed using a western blot imaging system (GE Amersham Imager 600, United States), and captured images were quantified using ImageJ software (NIH).

### Dual-Energy X-Ray Absorptiometry

Body BMD was assessed by dual-energy X-ray absorptiometry (DXA) (LU43616CN, GE Healthcare, Madison, WI, United States) using the small laboratory animals scan mode. Animals were anesthetized with an i.p. injection of sodium pentobarbital prior to scanning. Whole-body DXA assays were conducted at the end of the experiment. BMC and BMD from NOS2^–/–^ and WT rats were detected by DXA. All rats were coded, and the investigator was blinded to group allocation during the experiments. BMC and BMD were calculated automatically by a software package (enCore 2015; GE Healthcare).

### Histological Analysis

Tissues were fixed in 10% buffered formalin and embedded in paraffin. Tissue sections were obtained from subcutaneous white adipose tissue (S.C. WAT) and stained with hematoxylin-eosin (H&E). All samples were coded, and the investigator was blinded to the group allocation during the experiment.

### Statistical Analysis

For *in vitro* experiments, all results presented represent data collected from at least three independent experiments. Statistical analyses were performed using paired *t*-tests (2-tailed). Differences between groups *in vivo* were tested for statistical significance using the unpaired two-tailed Student’s *t*-test. Statistical tests were performed using GraphPad Prism version 7.0, and a *p*-value < 0.05 was considered statistically significant.

## Results

### Characteristics of BMSCs Derived From NOS2^–/–^ and WT SD Rats

**FIGURE 1:**
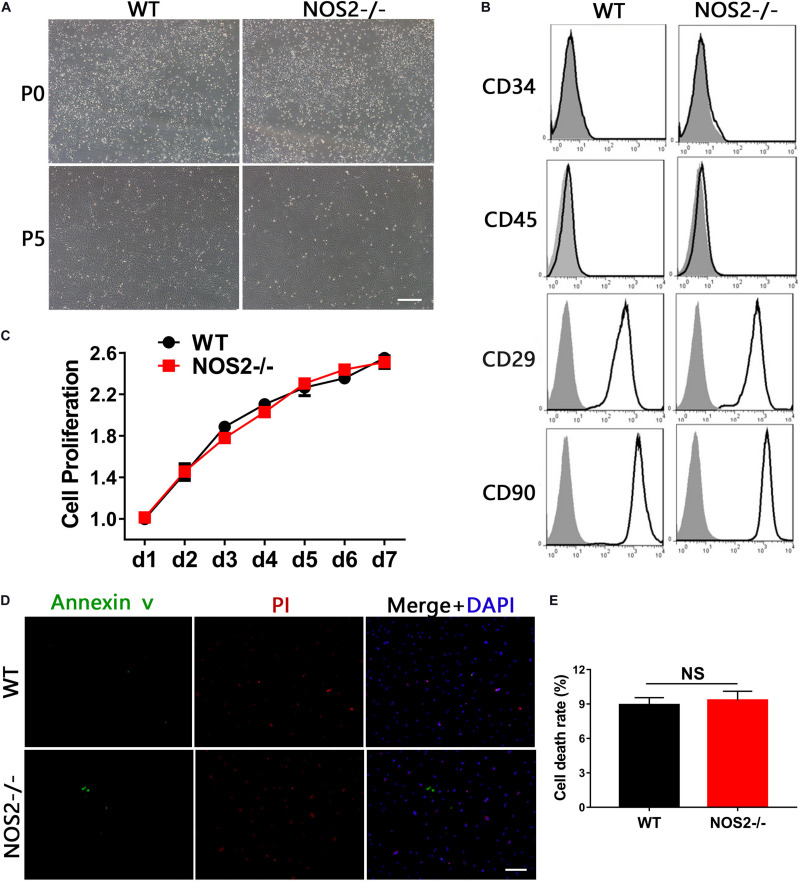
Characteristics of BMSCs from NOS2^–/–^ and WT SD rats. **(A)** The morphology of BMSCs was monitored under a microscope at passages 0 and 5; the scale bar indicates 200 μm. **(B)** The third passage of BMSCs derived from NOS2^–/–^ and WT rats were subjected to flow cytometry after staining with α-CD34, α-CD45, α-CD29 and α-CD90 (black line) or their corresponding isotype (shadowed). **(C)** Cell proliferation was analyzed by CCK8 assay. **(D,E)** BMSCs derived from NOS2^–/–^ and WT SD rats were cultured in serum-free medium for 48 h. Cell survival was analyzed using a LIVE/DEAD viability/cytotoxicity kit, and the scale bar indicates 100 μm. The results are expressed as the means ± SEM; N.S., not significant. *n* = 4.

Bone marrow-derived MSCs from NOS2^–/–^ and WT SD rats were easily obtained by adherent culture of BM cells. Isolated BMSCs exhibited fibroblast-like cell morphology and formed homogenous colonies (Figure 1A). Flow cytometry analysis revealed that BMSCs from both WT and NOS2^–/–^rats expressed the same panel of surface markers, including CD29 and CD90, but not the hematopoietic stem cell markers CD34 or CD45 (Figure 1B), indicating that NOS2 knockout may not alter the phenotype of BMSCs.

Furthermore, knockout of rat NOS2 did not alter the proliferative properties of BMSCs, which were verified by CCK8 assays (*p* = 0.49, Figure 1C). We additionally tested whether NOS2 knockout altered the rate of apoptosis of two types of MSCs. As shown in Figure 1D, culture under serum-deprived conditions for 48 h produced only a mild, non-significant increase in the death ratio that was similar to that found in NOS2-/- BMSCs (9.83 ± 0.75%) and WT BMSCs (8.72 ± 0.62%; *p* = 0.35) (Figure 1E). These results demonstrate that the morphology, phenotype, and proliferative and survival characteristics of rat MSCs with knockout of NOS2 showed no observable differences from those of WT rat MSCs.

### Immunosuppressive Functions of BMSCs From NOS2^–/–^ and WT SD Rats

**FIGURE 2:**
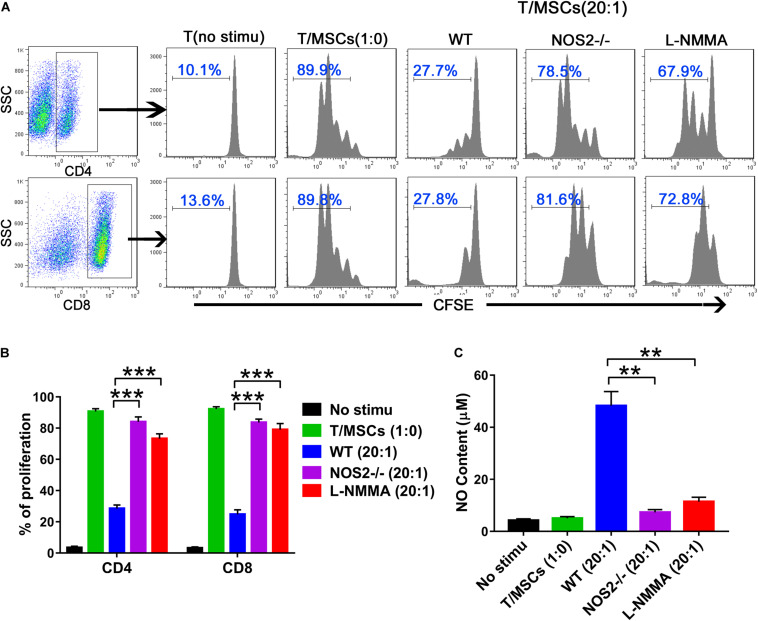
The immunosuppressive effects of NOS2^–/–^ and WT rats BMSCs on splenic T cells. Purified CD3 + T cells were stained with CFSE and cocultured with BMSCs (5.0 × 10^4^ cells per well in 24-well plates) at a ratio of 1:20 (MSC: T cells) for 96 h in the presence of α-CD3 and α-CD28 mAbs. The NOS2 inhibitor L-NMMA (1 mM) was added into the coculture system. **(A,B)** CFSE fluorescence intensity of CD4 + and CD8 + T cells was detected by flow cytometry. **(C)** NO production in T cells and activated T/MSCs cocultures was determined by Griess reagent. All results are expressed as the means ± SEM; **P < 0.01; ***P < 0.001; *n* = 3.

The immunosuppressive effects of MSCs on T cell proliferation were evaluated by co-culture of MSCs during T cell activation, which was rescued by a specific inhibitor of NOS (e.g., *N*^*G*^-monomethyl-L-arginine acetate salt, L-NMMA) (Ren et al., 2008). Thus, we cocultured BMSCs with purified T cells at graded ratios of 1:20 (MSC to T cells) for 4 days in the presence of α-CD3 and α-CD28, observing that T cell proliferation was completely blocked in the presence of WT BMSCs (Figure 2A). However, NOS2^–/–^ BMSCs or the presence of the NOS inhibitor L-NMMA failed to inhibit T cell proliferation (NOS2^–/–^ BMSCs, *p* < 0.001; L-NMMA, *p* < 0.001) (Figure 2B). This failure seems to be corresponding to NO production in the supernatant of BMSC-T cell cocultures. High levels of NO were detected in WT BMSCs coculture system, whereas little NO was detected in NOS2^–/–^ BMSCs, and L-NMMA cocultures (NOS2^–/–^ BMSCs, *p* < 0.01; L-NMMA, *p* < 0.01) (Figure 2C). These results strongly suggest that rat MSC-mediated immunosuppression depends on NO production or NOS2 activity.

### Differentiation Potential of NOS2^–/–^ and WT MSCs

**FIGURE 3:**
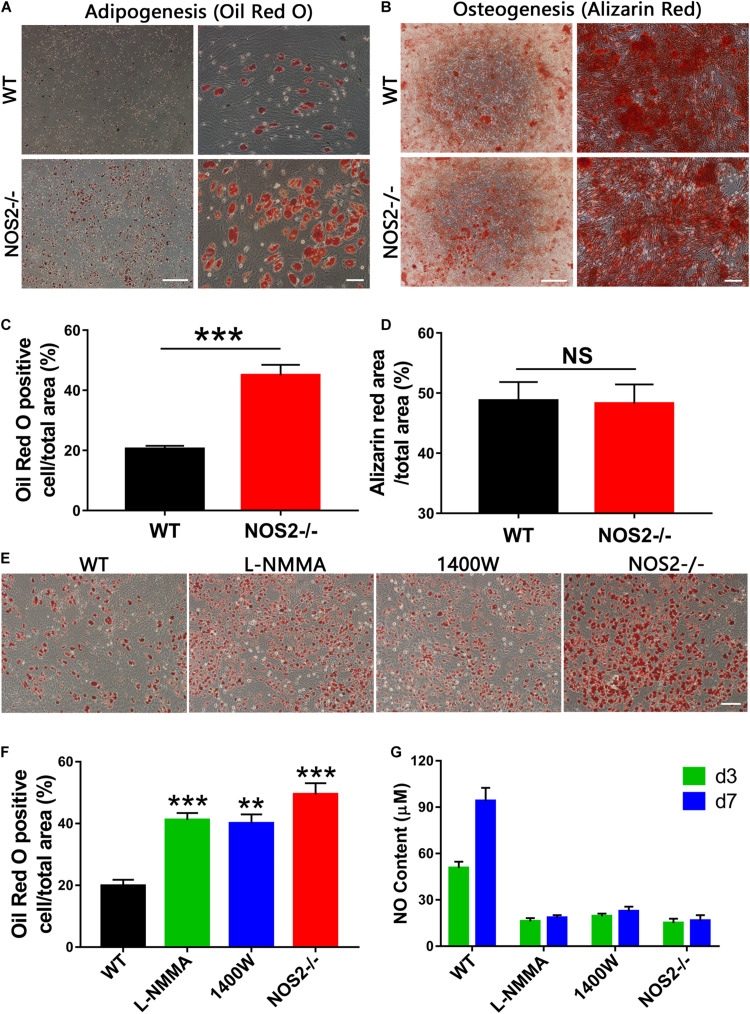
Differentiation capacity of MSCs from NOS2^–/–^ and WT SD rats. **(A)** BMSCs from NOS2^–/–^ and WT rats at passage 3 were induced to differentiate into adipocytes and osteoblasts in differential medium and were detected as described in the Materials and Methods. **(A,B)** Adipocyte and osteoblast differentiation indicated by Oil Red O staining **(A)** and Alizarin red S staining **(B)**, respectively; the scale bars indicate 200 μm (left) and 50 μm (right). **(C,D)** Statistical analyses of panels **(A,B)** (*n* = 4), respectively. **(E)** Adipocyte differentiation indicated by Oil Red O staining. L-NMMA (1 mM) and 1400 W (100 μM) were added to adipogenic differentiation conditions of WT BMSCs; the scale bar indicates 100 μm. **(F)** Statistical analyses of panel **(E)** (*n* = 4). **(G)** NO content of cell supernatants in panel **(E)** was detected by Griess reagent. All results are expressed as the means ± SEM; **P < 0.01; ***P < 0.001; N.S., not significant. *n* = 4.

To define the potential of MSCs to differentiate into adipocytes and osteoblasts, BMSCs from NOS2^–/–^ and WT SD rats were cultured under their respective culture conditions (AM A/B for 12 days and OM for 21 days). After staining with Oil Red O or Alizarin Red S, we found that both sets of BMSCs had successfully differentiated into adipocytes (Figure 3A) and osteoblasts (Figure 3B). The multipotency of these cells further confirmed their identity as MSCs. However, to our surprise, quantitative analysis revealed that there was a significantly higher proportion of adipogenically differentiated BMSCs in NOS2^–/–^ samples than in WT samples (*p* < 0.001, Figure 3C), while osteogenic differentiation was not altered between the groups (*p* = 0.91, Figure 3D). These findings were confirmed in AdMSCs, that is, MSCs from subcutaneous adipose tissue of NOS2^–/–^ and WT SD rats, as similar characteristics were observed for these cells (Supplementary Figure 1). Furthermore, administration of L-NMMA (NOS inhibitor) and 1,400 W {N-[3-(aminomethyl)benzyl] acetamidine}, a highly selective NOS2 inhibitor] to adipogenic differentiation conditions resulted in decreases in both adipogenic capacity and NO production (Figures 3E–G). These results strongly suggest that NO from NOS2 plays an important role in the adipogenesis of rat MSCs.

### NOS2 Knockout Increases Expression of Adipogenesis-Related Genes

**FIGURE 4:**
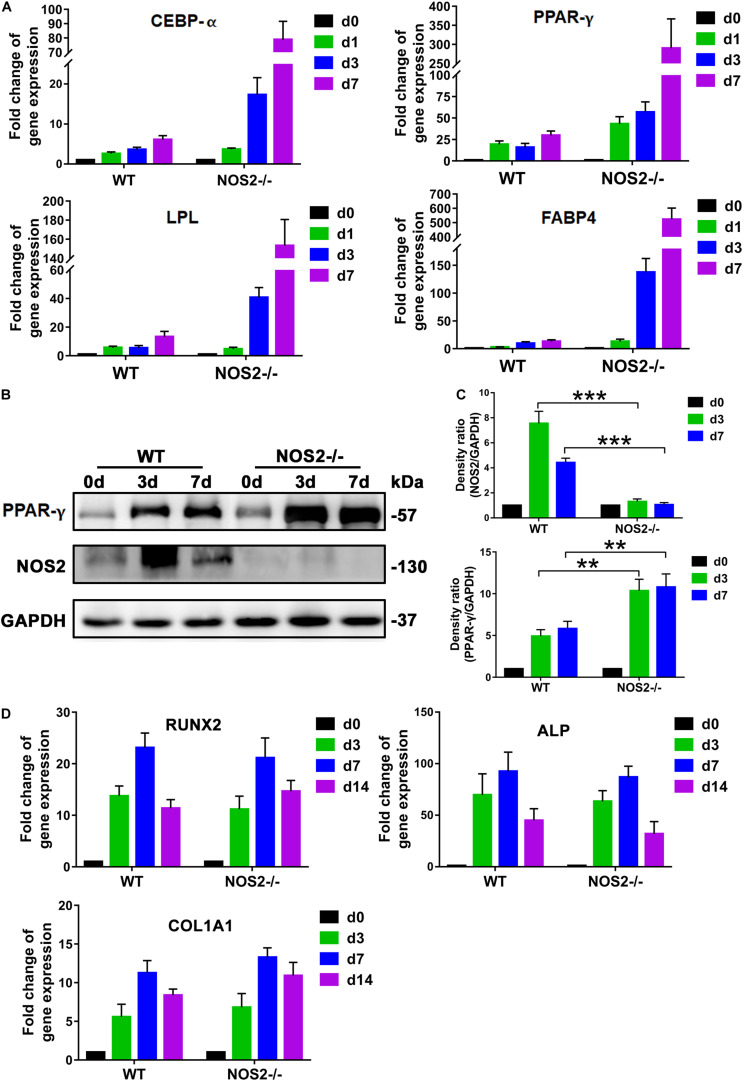
NOS2 knockout increases the expression of adipogenesis-related genes. BMSCs from NOS2^–/–^ and WT rats at passage 3 were induced to differentiate into adipocytes and osteoblasts and then collected to detect differentiation related genes. **(A)** qRT-PCR analyses of the expression of the transcription factors C/EBP-α and PPAR-γ and adipose differentiation-related genes LPL and FABP4 in MSCs from NOS2^–/–^ and WT rats on d 1, 3, and 7 after differentiation **(A)** compared to those of the controls without differentiation (d 0). **(B)** BMSCs were cultured in AM for d 0, 3, and 7 days, and western blotting was performed to analyze induction of PPAR-γ. **(C)** Density ratio of PPAR-γ or NOS2 and GAPDH in panel **(B)**. **(D)** BMSCs were cultured in OM for d 0, 3, 7, and 14 days, and osteogenesis differentiation-related genes in BMSCs were detected by qRT-PCR. All results are expressed as the means ± SEM; **P < 0.01; ***P < 0.001; N.S., not significant. *n* = 4.

During recent decades, a number of reports have revealed that adipogenesis is controlled by a complicated network of transcription factors, of which CCAAT-enhancer binding proteins (C/EBPs) and peroxisome proliferator-activated receptor gamma (PPAR-γ) play critical roles (Rosen et al., 2002; Farmer, 2006). In our study, Quantitative Real-Time PCR (qRT-PCR) analysis of the major regulators and markers of differentiation, including C/EBP-α, PPAR-γ, lipoprotein lipase (LPL), and fatty acid-binding protein 4 (FABP4), exhibited marked differences between NOS2^–/–^ and WT rat BMSCs during the differentiation period of 7 days, particularly the transcription factor PPAR-γ (Figure 4A). Therefore, protein expression levels of PPAR-γ and NOS2 during the period of differentiation were further investigated by western blot. As shown in Figures 4B,C, expression of PPAR-γ were greatly increased in NOS2^–/–^ BMSCs, and as expected, NOS2^–/–^ BMSCs failed to express the NOS2 protein. Furthermore, we also assessed the gene expression of major regulators and markers of osteogenic differentiation, including HOXA2/runt-related transcription factor 2 (RUNX2), alkaline phosphatase (ALP), and collagen type I alpha 1 chain (COL1A1). The results showed little difference between NOS2^–/–^ and WT BMSCs during the differentiation period of 14 days (Figure 4D). Therefore, our findings strongly suggest that the greater adipogenic potential in NOS2^–/–^ BMSCs is primarily due to the greater activation of adipogenic transcription factors than in WT BMSCs.

### Genetic Deletion of NOS2 Tends to Develop HFD-Induced Obesity in Rats

**FIGURE 5:**
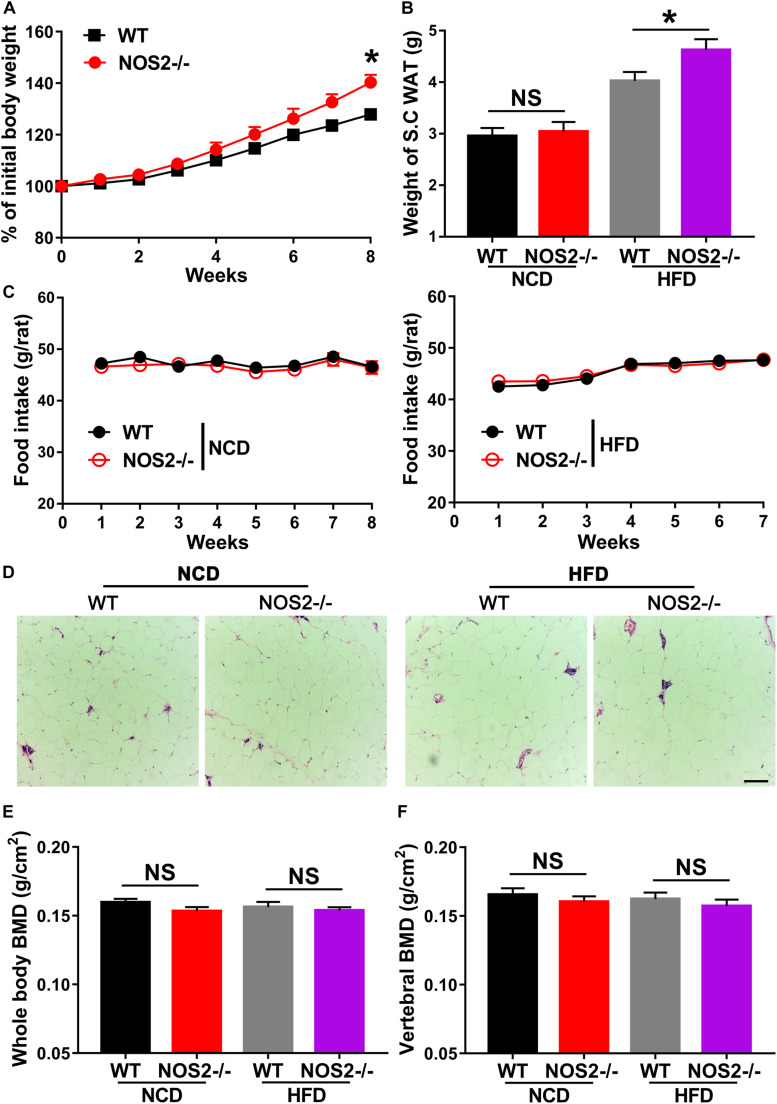
Genetic Deletion of NOS2 promotes HFD-Induced Obesity. Eight-week-old female WT and NOS2^–/–^ rats were fed either NCD or HFD for 8 weeks. **(A)** NOS2^–/–^ rats gained more weight compared to WT rats on HFD (*n* = 10). **(B,C)** Changes in S.C. WAT pads and food intake in WT and NOS2^–/–^ rats after 8 weeks of NCD or HFD feeding (*n* = 10). **(D)** Representative H&E staining images of S.C. WAT after 8 weeks of NCD or HFD feeding (*n* = 6), the scale bar indicates 100 μm. **(E,F)** Whole-body BMD **(E)** and vertebral BMD **(F)** evaluated by DXA after 8 weeks of NCD or HFD feeding (*n* = 6). All results are expressed as the means ± SEM; *P < 0.05; N.S., not significant.

Given the importance of NOS2 in the regulation of adipogenic differentiation and in disease, we investigated WT and NOS2^–/–^ rats under conditions of metabolic stress. WT and NOS2^–/–^ rats were fed a NCD or a HFD for 8 weeks to induce obesity. WT and NOS2^–/–^ rats had comparable weights on NCDs, but NOS2^–/–^ rats more easily became obese in response to the HFD (Figure 5A). The subcutaneous white adipose tissue (WAT) pads from NOS2^–/–^ rats were bigger than those in WT rats on a HFD (Figure 5B), while no considerable difference was observed in food intake between WT and NOS2^–/–^rats on either NCD or HFD (Figure 5C). Furthermore, H&E staining of histological sections of WAT from WT and NOS2^–/–^ rats showed no differences in WAT organization (Figure 5D).

To determine whether knockout of rat NOS2 affects osteoblast phenotype in response to a HFD, we analyzed BMD and BMC in rat whole body using DXA. As shown in Figures 5E,F, NOS2^–/–^ rats displayed almost no changes in whole body BMD and vertebral BMD compared to WT rats on either NCD or HFD. Whole body BMC and vertebral BMC were also unchanged in WT and NOS2^–/–^ rats (Supplementary Figure 2). Taken together, our results clearly indicate that knockout of rat NOS2 promotes adipogenesis without affecting osteogenesis *in vivo*, consistent with our *in vitro* findings.

### NOS2 Knockout Promotes Adipogenic Differentiation of Rat BMSCs Through the JAK2/STAT3 Signaling Pathway

**FIGURE 6:**
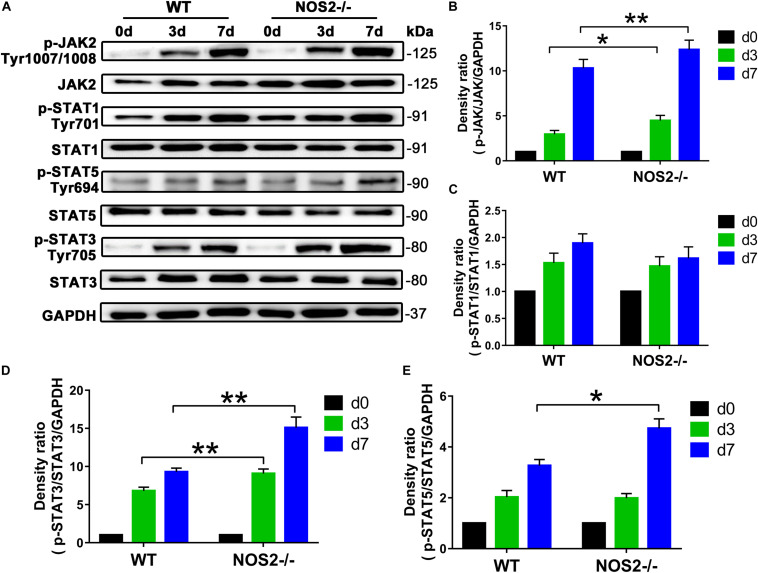
Knockout of NOS2 promotes rat MSC adipocyte differentiation through signal transducer and activator of transcription (STAT)3 and JAK signaling. BMSCs from NOS2^–/–^ and WT rats at passage 3 were induced to differentiate into adipocytes and were collected to detect the expression of proteins in the JAK/STAT signaling pathway at the indicated time points. The protein levels of p-JAK2, JAK2, p-STAT1, –3, and –5 and STAT1, –3, and –5 in NOS2^–/–^ and WT rat BMSCs were assessed by western blotting. **(A)** Representative western blots are shown. **(B–E)** Density ratios of p-JAK2 and JAK2, p-STAT1 and STAT1, p-STAT3 and STAT3, and p-STAT5 and STAT5. Data are normalized to GAPDH. All results are expressed as the means ± SEM; *P < 0.05; **P < 0.01; *n* = 4.

Since the JAK/STAT signaling pathway is related to cell proliferation and differentiation (Richard and Stephens, 2014), we further investigated whether this pathway is involved in the upregulated adipogenesis in NOS2^–/–^ rat MSCs. Western blot analysis showed that JAK activation (p-JAK2) was increased by 52.6 and 19.9% on days 3 and 7 after differentiation, respectively, compared to the WT BMSC group (d3, *p* < 0.05; d7, *p* < 0.01) (Figures 6A,B). The downstream protein p-STAT3, but not p-STAT1 or p-STAT5, was also increased by 33.4 and 62.5%, respectively (d3, *p* < 0.01; d7, *p* < 0.01) (Figures 6A–E).

**FIGURE 7:**
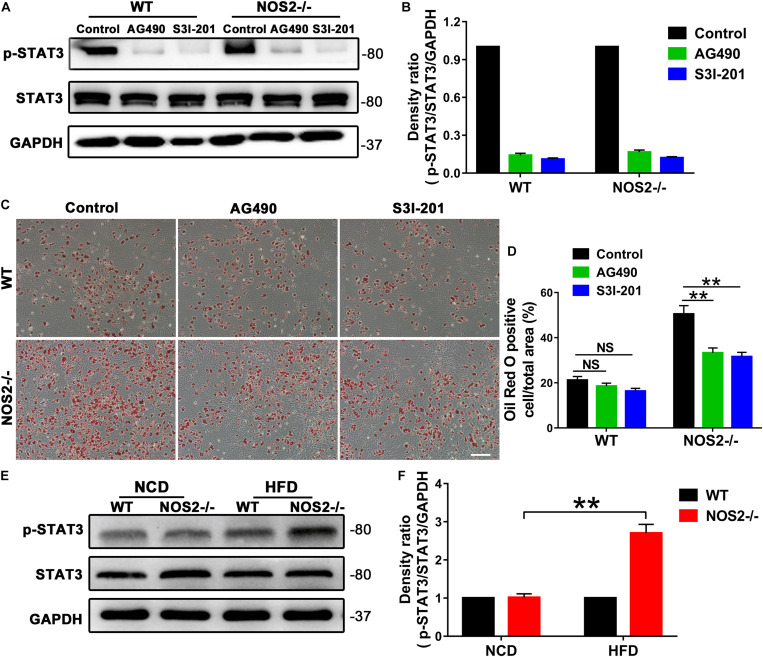
AG490 and S3I-201 inhibit STAT3 activation and adipocyte differentiation in NOS2^–/–^ rat MSCs. BMSCs from NOS2^–/–^ rats at passage 3 were induced to differentiate into adipocytes in the presence or absence of AG490 (10 μM) and S3I-201 (20 μM) and were collected to detect p-STAT3 and STAT3 expression levels **(A,B)** and adipocyte differentiation **(C,D)** on d 12 after differentiation. **(E)** Representative western blots are shown in WAT from WT and NOS2^–/–^ SD rats after HFD feeding. **(F)** Density ratios of p-STAT3 and STAT3. The scale bar indicates 100 μm. All results are expressed as the means ± SEM; **P < 0.01; *n* = 4.

To determine the role of STAT3 in rat MSC adipocyte differentiation, we utilized the JAK2 -specific inhibitor AG490 and the STAT3-selective inhibitor S3I-201 (NSC 74859) in NOS2^–/–^ and WT BMSC adipogenesis induction. As shown in Figures 7A–D, both AG490 and S3I-201 inhibited STAT3 activation (Figures 7A,B) and NOS2^–/–^ BMSC adipogenesis (for AG490, *p* < 0.01; for S3I-201, *p* < 0.01) (Figures 7C,D) after 12 days’ differentiation. In contrast, the two inhibitors had no significant effect on WT BMSC adipogenesis (Figures 7C,D). Most importantly, western blotting also revealed increased expression of p-STAT3 protein in NOS2^–/–^ rat WAT with HFD feeding (Figures 7E,F). Our findings strongly suggest that the greater adipogenic potential of NOS2^–/–^ BMSCs was primarily due to greater activation of the JAK2/STAT3 signaling pathway than occurs in WT BMSCs.

## Discussion

In recent years, much attention has been given to NO as a key regulator of cell differentiation. The inducible synthase of NO, NOS2, was found to regulate the differentiation and function of many immune cells, including T cells (Lee et al., 2011; Niedbala et al., 2011; Jianjun et al., 2013; Obermajer et al., 2013; Garcia-Ortiz and Serrador, 2018). Here, our data confirmed that BMSCs derived from NOS2^–/–^ rats showed a clear immunosuppressive effect on T cell proliferation. These findings are consistent with the specific NOS inhibitor L-NMMA (Su et al., 2014) and are further supported by studies in NOS2-knockout mice, which also exhibited abolished cytokine-induced immunosuppression in MSCs (Ren et al., 2008). In mouse, low concentrations of NO promote Th1 differentiation, whereas high concentrations of NO from NOS2 promote Th2 cell differentiation and inhibit Th17 differentiation (Lee et al., 2011; Niedbala et al., 2011; Jianjun et al., 2013). In contrast, NO produced by NOS2 in activated CD4 + T cells is necessary for the induction and stability of human Th17 cells (Obermajer et al., 2013). In a mouse muscle injury model, NO released by infiltrating NOS2-positive macrophages was necessary for the proliferation and differentiation of myogenic precursor cells (Rigamonti et al., 2013). Indeed, various studies have shown that the differentiation and functional regulation of many immune cells, including T cells, macrophages, and mature dendritic cells (mDCs), by NOS2 are mediated *via* nitration of key molecules in transcriptional or signaling pathways (Mao et al., 2013; Mishra et al., 2013; Bogdan, 2015). Our findings once again highlighting the role of NOS2 and MSCs in the regulation of immunity.

In addition to their roles in immunoregulation, MSCs are fibroblast-like multipotent cells that have the ability to differentiate into osteoblasts, adipocytes, chondrocytes, and thus a powerful tools for tissue engineering, regenerative medicine and also drug delivery (Pittenger et al., 1999; Gjorgieva et al., 2013; Ackova et al., 2016; Chen et al., 2016). As osteoblast differentiation was not altered in our NOS2^–/–^ rat BMSCs, we considered that NOS2 may not play an essential role in rat MSC osteogenic differentiation. However, many studies on total NO have observed its effects on the physical activities of bone, including bone development, bone healing, and bone resorption (Collin-Osdoby et al., 1995; Chow, 2000; Zhang et al., 2016). Recently, Yang et al. (2018) reported that NO balances osteoblast and adipocyte lineage differentiation *via* the JNK/MAPK signaling pathway in periodontal ligament stem cells. The contrast in these findings may be due to the source of NO, as these results did not distinguish among the activities of NOS1, NOS2 or NOS3, any of which could be the true regulator(s) of osteogenic differentiation. A study on mouse did support this explanation, as NOS1^–/–^ mice exhibited profound abnormalities in bone formation, and their osteoblasts showed significant delays in differentiation (Van’T et al., 2004). Importantly, a study on NOS2^–/–^ mouse also showed decreased osteoblast growth, and the resulting osteoblasts covered a smaller culture dish area and generated fewer resorption pits (Herrera et al., 2011). Since mouse NOS2 produces significantly lower NO levels than rat NOS2 (Zhao et al., 2013), and NOS1 in both mouse and rat produces even lower levels (Li et al., 2012), we considered only an appropriate amount of NO may influence osteogenesis. On the other hand, NOS2 may only influence the growth of osteoblasts, which was not observed in our experiments. Therefore, we conclude that NOS2 may not affect osteogenic differentiation of MSCs.

A theoretical inverse relationship exists between osteogenic and adipogenic lineage commitment and differentiation such that differentiation toward adipogenesis occurs at the expense of osteogenesis. However, we found this may not be true in the present study, as NOS2^–/–^ rat BMSCs showed significantly increased adipogenesis by Oil Red O staining with osteoblast differentiation remaining unaltered (Figure 3). Our finding on adipogenesis is supported by previous studies on the production of NO promoting adipocyte differentiation (Yan et al., 2002; Engeli et al., 2004; Hemmrich et al., 2007), although other observations have suggested that NO exerts the opposite effect on adipogenesis (Kawachi et al., 2007; Cordani et al., 2014; Yang et al., 2018). These conflicting findings may be due to the effect of a less specific inhibitor, the use of different MSCs, or the assaying of different NO sources (endogenous or exogenous), since NO is also generated by NOS1 and NOS3, and not just NOS2, in these cells. These specificity issues may be solved by using our model of NOS2 knockout rats. Therefore, we propose that NOS2 and its product NO are essential regulators suppressing excessive adipogenesis differentiation in rat MSCs. This is an interesting indication that perhaps when losing NOS2/NO, MSCs tends to switch their role from immunomodulation (specific inhibition of T cell proliferation) to energy storage (adipocyte differentiation).

A cascade of sequential transcriptional regulatory bursts underpins adipogenesis, including C/EBP-α and PPAR-γ, which are major late transcription factors (Rosen et al., 2002; Farmer, 2006). PPAR-γ is a ligand-activated nuclear receptor and is indispensable for adipocyte differentiation both *in vitro* and *in vivo* (Kawai and Rosen, 2010). In our study, qRT-PCR and western blot results revealed that PPAR-γ expression was significantly increased in NOS2^–/–^ MSCs compared to WT MSCs, suggesting a role for PPAR-γ in connecting NOS2 depletion and adipogenesis. Such a connection is also suggested by a few reports that PPAR-γ regulates human mesenchymal lineage allocation, favoring adipocyte over osteoblast development (Yu et al., 2012). Lee et al. (2010) reported anti-adipogenesis by 6-thioinosine is mediated by downregulation of PPAR-γ through JNK-dependent upregulation of NOS2. On the other hand, our findings also suggest that PPAR-γ may not influence osteogenesis. Consistent with our data, a study in human MSCs observed that PPAR-γ RNAi had no obvious effect on osteogenesis under permissive conditions (Bruedigam et al., 2008). These similarities between human and rat models may indicate the potential for using rat models to address human questions.

In addition to PPAR-γ, STAT3 may directly regulate adipogenic differentiation of MSCs. STAT3 belongs to the STAT (signal transducers and activators of transcription) signaling pathway family, which is a common pathway for signal transduction to regulate cytokines that affect several physiological processes, including cell proliferation, differentiation, apoptosis, and interactions with other signaling pathways (Stark and Darnell, 2012). Many studies on development of adipocytes from preadipocytes have revealed that STAT-mediated gene expression and modifications are cell-type specific. Transgenic knockout studies have also shown critical roles for every member of the STAT family (Richard and Stephens, 2014). STAT1, STAT3, and STAT5 are critical for adipogenesis (Zhao and Stephens, 2013). Our data demonstrated that STAT3 proteins from NOS2^–/–^ BMSCs are tyrosine phosphorylated (activated form) during the initiation of adipocyte differentiation (Figure 4), and this activation in NOS2^–/–^ BMSCs preceded the corresponding increase in WT BMSCs. Furthermore, deactivation of STAT3 by an inhibitor suppressed adipocyte differentiation in NOS2^–/–^ BMSCs. The clear involvement of the STAT3 pathway in adipocyte differentiation is consistent with the knowledge of the JAK2/STAT3 pathway’s involvement in early adipogenesis through regulation of C/EBP-β transcription (Zhang et al., 2011). Interestingly, a connection between STAT3 and NOS2 has been previously reported in glial proliferation and transformation (Puram et al., 2012), as well as in human macrophages in response to *Mycobacterium tuberculosis* infection (Zhou et al., 2019). Therefore, our finding that STAT3 expression was strongly upregulated during adipocyte differentiation in NOS2^–/–^ BMSCs suggests that STAT3 is a key factor in adipogenic differentiation.

Another possible route by which NO favors adipogenic differentiation may be its role in regulating lipid metabolism. Pioneering studies revealed the expression and activity of NOS2 in adipose tissue long ago (Ribiere et al., 1996) and verified an NO-mediated effect on lipolysis regulation both *in vitro* and *in vivo* (Gaudiot et al., 1998; Adam et al., 1999). The inhibitory action of NO on basal lipolysis, shown with *in vitro* chemical NO donors, may function *via* the inhibition of adenylyl cyclase (AC) and protein kinase A (PKA) (Adam et al., 1999; Klatt et al., 2000). NOS2 is a negative modulator of lipolysis *via* an oxidative signaling pathway upstream of cAMP production (Penfornis and Marette, 2005). In adipose tissue, its fuel-buffering capacity is also dependent on the physiological levels of NO (Jankovic et al., 2017) as a small redox molecule. These results indicate that physiological levels of NOS2/NO play a pivotal role in maintaining healthy metabolic function of adipose tissue. The existence of NOS2 may ensure MSC’s adipogenic differentiate without excessive differentiation or no differentiation. Of course, the pathogenesis of obesity is far more complex than just lipid accumulation and involves interactions among many cell types (Choe et al., 2016). Under conditions of metabolic stress, lipid accumulation in NOS2-/- rat maybe develop into a variety of metabolic syndrome, such as insulin resistance (Jiang et al., 2011), type 2 diabetes (Kopelman, 2000), vascular pathology (O’Rourke et al., 2011) and hepatic steatosis (Festi et al., 2004). Therefore, whether and how these lipid metabolic changes due to NO depletion alter the tendency toward MSC differentiation into adipocytes would be very interesting to address in future studies.

Moreover, NO may influence cells, tissues/organs, and consequently the whole body in various ways. In subcutaneous WAT, NO produced by NOS2 in obese individuals may impair insulin-stimulated glucose uptakes or contribute to decreased lipolytic rates, contributing to increased lipid storage (Penfornis and Marette, 2005). In liver, NO decreases hepatic lipogenesis through its actions on coenzyme A, forming a metabolically inactive compound (Greif et al., 2002). In skeletal muscle, NO also decreases lipogenesis *via* the activation of AMP-activated protein kinase, which is associated with increased fatty acid oxidation during exercise (Winder and Hardie, 1996). Therefore, it is not surprising to find that NO is depleted in NOS3-knockout mice and that these mice exhibited increased abdominal fat mass, dyslipidemia, and insulin resistance (Nisoli et al., 2003). Therefore, our findings regarding the effects of NO on adipogenesis greatly highlight its roles in the physiology of adipogenesis.

In addition, NOS2 activity has been found relevant to cellular senescence in various cell types (Ropelle et al., 2013; Katsuumi et al., 2018). Aging BMSCs display a shift in differentiation ratio of less osteoblasts and more adipocytes (Qadir et al., 2020), which is interestingly a bit similar with our findings in NOS2-/- MSCs. A further clarification of both processes may reveal the potential of sharing underlying mechanisms to some extent.

In summary, knockout of NOS2 impairs the function of MSCs with respect to regulation of immunity by enhancing their capacity for adipogenesis, but not osteogenesis, *via* PPAR-γ and STAT3.

## Data Availability Statement

The original contributions presented in the study are included in the article/Supplementary Material, further inquiries can be directed to the corresponding author/s.

## Ethics Statement

The animal study was reviewed and approved by the Experimental Animal Ethics Committee of Guangzhou Medical University.

## Author Contributions

AQ was responsible for isolating and culturing rat MSCs, participated in the design of the study and analysis of the data, and drafted the manuscript. SC and PW performed major experiments and analyzed data. XH was in charge for the detection and analysis of flow cytometry samples. YZ performed the qRT-PCR experiment. LL and L-RD were in charge of detection and analysis of osteoblast phenotype or adipocytic phenotype from rats. D-HL participated in the design of the study and drafted the manuscript. LD performed the detection and analysis of HE staining. XY and AX supervised the experiments and secured grants. All authors read and approved the final version of the manuscript.

## Conflict of Interest

The authors declare that the research was conducted in the absence of any commercial or financial relationships that could be construed as a potential conflict of interest.
