# Supplementary material for: Knockout of NOS2 Promotes Adipogenic Differentiation of Rat MSCs by Enhancing Activation of JAK/STAT3 Signaling
Source: Front Cell Dev Biol. 2021 Mar 19;9:638518. doi: 10.3389/fcell.2021.638518 (PMC8017136; doi:10.3389/fcell.2021.638518)
Supplement: Supplementary file 1 [file Data_Sheet_1.docx]

**Supplementary Materials**

Supplementary fig. 1.


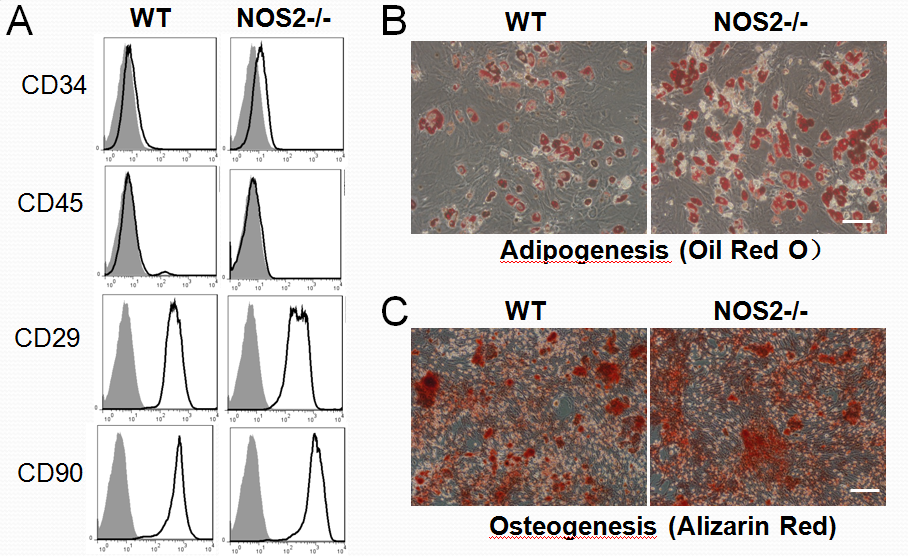
The phenotype and differentiation capacity of AdMSCs from NOS2-/- and WT SD rats. (A) Third passage adipose-derived MSCs (AdMSCs) derived from NOS2-/- and WT rats were subjected to flow cytometry after staining with α-CD34, α-CD45, α-CD29 and α-CD90 (black line) or their corresponding isotype (shadowed). (B and C) Adipocyte and osteoblast differentiation indicated by oil red O staining (A) and Alizarin red S staining (B), respectively; the scale bar represents 50 μm. (D and E) Statistical analyses of the B and C graphs (n = 4), respectively. Indicated values are means ± SEM. *p< 0.05, **p< 0.01, compared with the controls. Abbreviations: N.S., not signiﬁcant.

**C**

Supplementary Fig. 2.

BMC change in WT and NOS2-/- rats after NCD or HFD feeding.

(A and B) Eight-week-old female WT and NOS2-/- rats were fed either NCD or HFD for 8 weeks. The whole body BMC (A) and vertebral BMC (B) evaluated by DXA (n = 6/group).


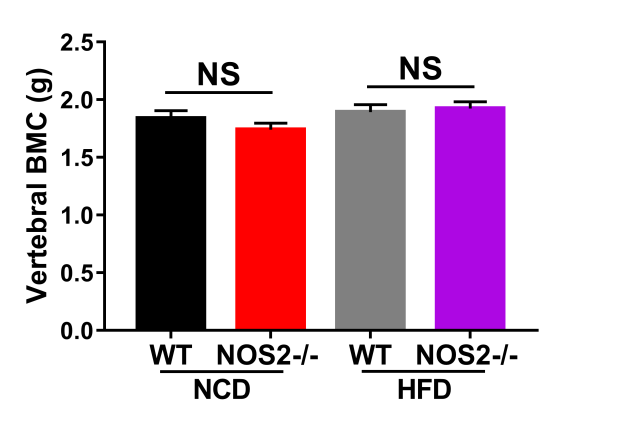

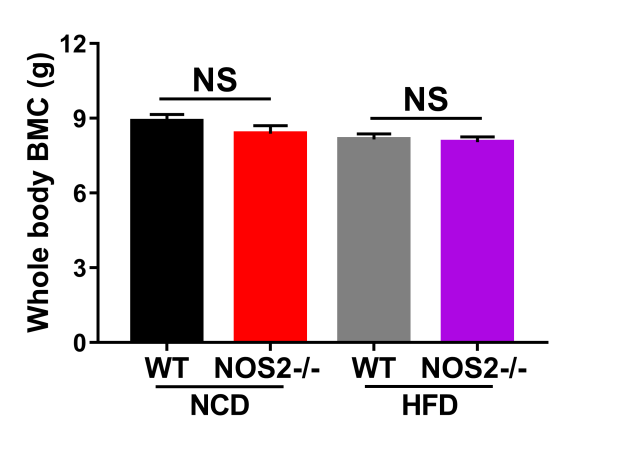


**A B**

**Supplementary Table1. Oligonucleotides used in this study.**

| Assay | Target Gene | Sequence (5′-3′) |
| --- | --- | --- |
| Quantitative real-time  primers | GAPDH | F: TTACCAGGGCTGCCTTCTCTTG |
|  |  | R: GATCTCGCTCCTGGAAGATGG |
|  | PPAR-γ | F: TGCTGGTGATCAGAAGGCTG |
|  |  | R: TGTGTCAACCATGGTAATTTCAGT |
|  | C/EBP-α | F: TTTCGTAACCGTCGCTCCTC |
|  |  | R: TTCACATGTACCTGCGCCTC |
|  | FABP4 | F: TTTCCTTCAAACTGGGCGTG |
|  |  | R: TTTCATGACACATTCCACCACC |
|  | LPL | F: TCCAGCCAGGATGCAACATT |
|  |  | R: AACTCAGGCAGAGCCCTTTC |
|  | ALP | F: ATAGAGCTGGTGACAAGGGTG |
|  |  | R: ACGCTCTTCCCCGTCTTACA |
|  | RUNX2 | F: GCTTCATTCGCCTCACAAACA |
|  |  | R: CTTGCAGCCTTAAATGACTCGG |
|  | COL1A1 | F: CATGGCCTCTGCAACAAATCC |
|  |  | R: ACCAGAAATTCCTTCCCACCC |
